# Unilateral Intervention in the Sinuses of Rabbits Induces Bilateral Inflammatory and Microbial Changes

**DOI:** 10.3389/fcimb.2021.585625

**Published:** 2021-09-14

**Authors:** Christian A. Lux, James J. Johnston, Sharon Waldvogel-Thurlow, Camila Dassi, Richard G. Douglas, Do-Yeon Cho, Michael W. Taylor, Kristi Biswas

**Affiliations:** ^1^School of Biological Sciences, University of Auckland, Auckland, New Zealand; ^2^Department of Surgery, School of Medicine, University of Auckland, Auckland, New Zealand; ^3^Department of Otorhinolaryngology, Pontifícia Universidade Católica do Paraná (PUCPR), Curitiba, Brazil; ^4^Department of Otolaryngology-Head and Neck Surgery, University of Alabama at Birmingham and Veteran Affairs Medical Center, Birmingham, AL, United States

**Keywords:** sinusitis, chronic rhinosinusitis, inflammation, microbiota, dysbiosis, rabbit model, animal model

## Abstract

**Background:**

Chronic rhinosinusitis (CRS) is a globally prevalent inflammatory condition of the paranasal sinuses which severely impairs patients’ quality of life. An animal model of unilateral sinusitis by transient sinus occlusion has been described previously in rabbits. The aim of this study was to characterise the sinusitis rabbit model by investigating temporal and bilateral changes in the bacterial community and mucosal inflammation.

**Methods:**

Development of sinusitis was achieved by endoscopically placing Merocel*^®^*, a sterile nasal packing material, in the left middle meatus of six New Zealand white rabbits for four weeks. After a total period of 14 weeks, rabbits were assessed for sinusitis by endoscopic examination, magnetic resonance imaging (MRI) and histology. Swabs from the left and right middle meatus were obtained for bacterial community analysis at three time points (week 0, week 4, week 14) during the study.

**Results:**

Endoscopic evaluation showed unilateral inflammation in all animals examined after the 4-week blocking period and at week 14. Notably, inflammatory changes were also seen in the contralateral sinus of all animals at week 4. MRI images demonstrated unilateral sinus opacification at week 4 in two rabbits, and partial unilateral sinus opacification at week 14 in one rabbit only. Histological analyses revealed substantial spatial heterogeneity of mucosal inflammation with inconsistent findings across all animals. No significant differences in mucosal inflammatory markers (such as goblet cell hyperplasia, epithelial denudation and oedema) could be identified between nostrils at week 14. The bacterial community in the rabbit sinuses was heavily dominated by *Helicobacter* at week 0 (baseline). At the end of the blocking period (week 4), bacterial alpha and beta diversity were significantly increased in both nostrils. The bacterial community composition at week 14 had primarily returned to baseline, reflecting the endoscopic and radiological results.

**Conclusion:**

This study reaffirmed the ability for development of sinusitis without inoculation of any pathogens in a rabbit model. We were able to demonstrate bilateral sinonasal mucosal inflammation, by inducing unilateral sinus blockage, which resulted in significant changes to the sinonasal bacterial community. These findings may explain some of the clinical observations seen in CRS and warrant further research to reveal potential implications for its therapeutic management.

## Introduction

The pathogenesis of chronic rhinosinusitis (CRS) has been under intense research scrutiny, with several hypotheses put forward. Many studies suggest that the sinus microbial community plays a role in the pathobiology of CRS (Abreu et al., 2012; Boase et al., 2013; Biswas et al., 2015; Paramasivan et al., 2020), with bacterial dysbiosis (a deleterious shift in the microbiota) being reported (Hoggard et al., 2017; Chalermwatanachai et al., 2018). In addition, host immunity is considered a contributor to chronic mucosal inflammation (Van Crombruggen et al., 2011; Fokkens et al., 2020). Increased numbers of T and B lymphocytes and macrophages in the sinus mucosal tissue were identified as characteristic of CRS (Biswas et al., 2017; Yin et al., 2018) and interleukins, such as IL-5 and IL-13, play a role in the initial inflammation and its progression in this disease, especially with nasal polyposis (Kato, 2015; Jiao et al., 2016; Bachert et al., 2018; Chowdhury et al., 2019). The complex interplay between the immune response, sinonasal microbial community, medical treatment and sinus epithelial integrity complicates the investigation of underlying mechanisms in the development of CRS. The high inter-individual variation of disease severity and observed symptoms, coupled with the difficulty in controlling antecedent medical interventions and lifestyle components such as smoking and diet, make it challenging to gain insights from clinical studies.

Animal models can help overcome many of these issues and several species have been used successfully to study the pathophysiology of CRS (London and Lane, 2016; Shin, 2016; Lux et al., 2019). Animal models are particularly helpful when studying the efficacy of novel treatments (Chiu et al., 2007; Tamashiro et al., 2009; Jia et al., 2017; Yoruk et al., 2017). In a recent CRS-focused review of animal models for the study of chronic mucosal inflammation, rabbits were found to be a particularly suitable model in regards to animal handling and features of sinus anatomy and physiologic characteristics (Lux et al., 2019). Methods for inducing inflammation of the sinus mucosa include microbial inoculation (Abreu et al., 2012; Jia et al., 2014), administration of inflammatory agents (Gocea et al., 2013; Khalmuratova et al., 2016) and mechanical obstruction of the sinus ostia (Ha et al., 2007; Migliavacca et al., 2014; Cho et al., 2018), or a combination of these (Boase et al., 2011). An important pathway that underlies the pathogenesis and morbidity of CRS appears to be the impaired mucociliary clearance (Lusk, 1998). This results in the accumulation of hyper-viscous mucus, which restricts oxygen, promotes inflammation and supports microbial colonization (Cho et al., 2020). Ultimately, regardless of the etiology, a pathophysiological characteristic of CRS is inflammation of the sinonasal mucosa with obstruction of the sinus outflow tract (Lee and Lane, 2011). Therefore, sinus blockage, especially if applied reversibly, is advantageous when trying to mimic the physiological development of CRS. Temporary blockage has been shown to re-create the distinctive immune response and characteristic microbial community changes that are associated with the disease (Liang et al., 2008; Migliavacca et al., 2014). A rabbit model of unilateral CRS with excellent potential for microbiome research has been described previously (Cho et al., 2018).

In general, CRS is considered as a bilateral disease, although a subset of patients presents with single-sided symptoms (Fokkens et al., 2020). Kennedy and his colleagues previously demonstrated that a rabbit infected with *Pseudomonas aeruginosa* in the sinuses can show signs of inflammation at sites other than the site of primary infection (Perloff et al., 2000). However, to our limited knowledge, none of the animal models explored the development of bilateral sinonasal mucosal inflammation by inducing unilateral sinus blockage. In this study, we have attempted to induce CRS in rabbits by unilateral transient sinus obstruction for four weeks and investigated whether this could result in bilateral changes to the sinus bacterial communities and mucosal inflammation.

## Methods

### Animal Model

This study was approved by the University of Auckland Animal Ethics Committee (AEC# 001910). All animals used in this study were farm-sourced male and female New Zealand white rabbits (4-6 kg). A pilot study was conducted to establish study protocols and set up surgical procedures using three animals (data not shown). For the current study, six rabbits were used. Animals were kept at the animal facility at the University of Auckland. Rabbits were housed in individual cages with dry food and water provided *ad libitum*. Animal wellbeing was monitored throughout the study using an animal welfare scoring sheet based on the rabbit grimace scale. Before initiation, rabbits were acclimatised at the animal facility for at least one week.

Rabbits were anaesthetised using a combined injectable and inhalational approach. A mix of medetomidine (0.25 mg/kg, SVS Veterinary Supplies Ltd, Hamilton, New Zealand), ketamine (5 mg/kg, SVS) and buprenorphine (0.03 mg/kg, Onelink, Auckland, New Zealand) was given subcutaneously 30-45 min prior to placing the animals on oxygen for 1-2 min *via* facemask. At that point, the airway was accessed *via* a supraglottic airway device (V-Gel, Docsinnovent Ltd, London, United Kingdom). All rabbits were maintained (as needed) on 1-2.5% isoflurane at an oxygen flow rate of 0.8-1 L/min. Medetomidine was reversed with intramuscular atipamezole at 0.5-1 mg/kg. Rabbits were maintained on oxygen *via* facemask until aware of their surroundings and able to hold their head up. All rabbits were monitored continuously until they were able to hop adequately and showed an interest in food and water. Rabbits did not receive any other medication before or during the study.

### Induction of Unilateral Sinus Inflammation

**Figure 1:**
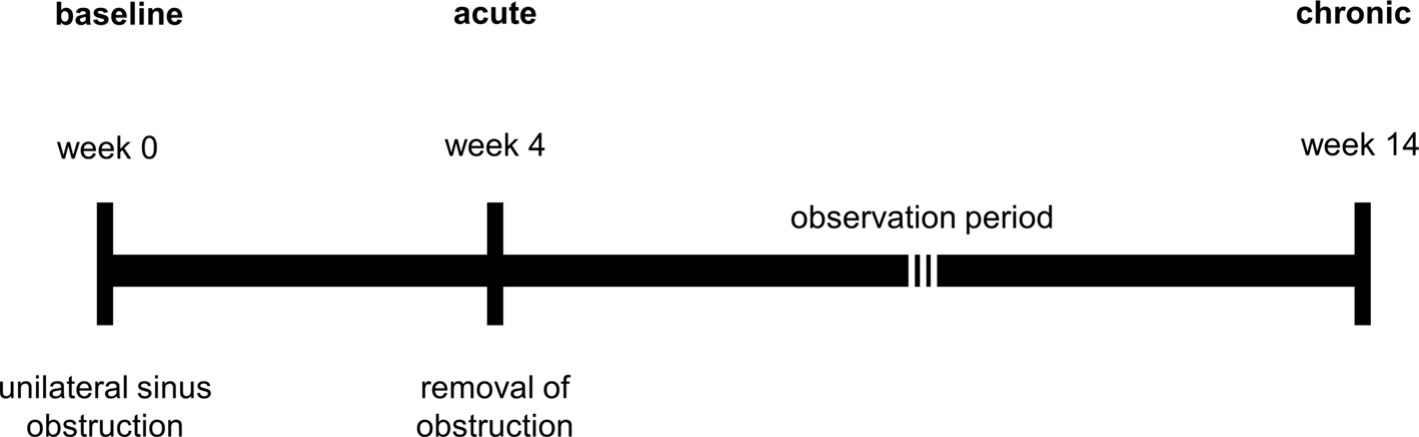
Timeline of model development.

Sinusitis was induced using a transnasal endoscopic technique as previously described by Cho et al. (2018). In brief, sterile nasal packing material (Merocel^®^, Medtronic, Auckland, New Zealand) was placed in the left middle meatus (unilateral) under endoscopic guidance to achieve complete obstruction of this sinus ostium. All procedures were carried out by an experienced otorhinolaryngology surgeon with the assistance of a veterinary anaesthetist and an animal welfare officer to ensure the best possible care for the rabbits during surgery. Our pilot study indicated that two weeks of sinus obstruction was insufficient to achieve significant and persistent sinus inflammation (data not shown). Accordingly, the blockage period was extended to four weeks. After the removal of Merocel^®^, all animals were observed for a further ten weeks, i.e., up to 14 weeks from the time point of obstruction. A timeline of the study is illustrated in **Figure 1**.

### Outcome Measures

Animals were examined for features of CRS throughout the study, and samples were collected at baseline (insertion of Merocel*^®^*), during the acute phase (Merocel*^®^* removal) and the chronic phase (culling of the animal). Examination and sample collection at each time point included endoscopic scoring and sinus swab collection from both nostrils for microbiota analysis. Additional MRI scans at the acute and chronic time points were performed. Finally, the rabbit snout was collected for histological analysis at the end of the study.

#### Endoscopic Score Grading for Sinus Inflammation

A 1.9 mm endoscope (HOPKINS^®^ Straight Forward Telescope 0°, Karl Storz, Tuttlingen, Germany) was used to examine the nasal cavities as previously described (Cho et al., 2017). A scale of 0 to 4 was used to grade inflammation with 0 representing normal, 1 mild inflammation, 2 moderate inflammation, 3 severe inflammation and 4 severe inflammation with ulceration or polyps and luminal opacification (Marks, 1997).

#### Magnetic Resonance Imaging

MRI scanning was performed using a 3T Skyra MRI system (Siemens Healthineers, Erlangen, Germany), with a 15 cm diameter 16-channel transmit-receive radiofrequency coil. Both coronal and transverse images, each with 30 contiguous 1.5 mm thick slices, were acquired using a T2-weighted turbo spin echo sequence so that all sinus cavities were covered and could be examined for sinus opacification. The images had a 120 x 120 mm field of view, acquired with a 320 x 256 matrix, reconstructed to give an in-plane resolution of 0.4 x 0.4 mm.

#### Histological Analysis

Animals were euthanised by the administration of an overdose of pentobarbital (SVS). After death was confirmed, the rabbits’ snouts were removed from the heads by making two intersecting cuts using an electric saw. Tissue samples were placed in Carnoy’s fixative (60% ethanol, 30% chloroform, and 10% glacial acetic acid) immediately after harvesting and incubated for 24 h. Snouts were decalcified subsequently in RDO Rapid Decalcifier (Apex Engineering, Illinois, USA). Decalcified snouts were then separated into sections as previously described (Pereira et al., 2011) and embedded in paraffin. Sections were stained with haematoxylin and eosin (H&E) to assess the structural integrity of the epithelium and submucosa, as well as to document immune cell infiltration. In addition, Periodic Acid – Schiff (PAS) stain was used to detect mucin-producing cells and structures (goblet cells and submucosal glands). The evaluation of tissue samples was conducted by two independent examiners and includes qualitative observation of inflammatory signs as well a semi-quantitative analysis. To quantify inflammatory markers and mucosal integrity in all animals, a set of parameters, including epithelial hypertrophy and thickness of the base membrane and submucosa (**Table S1**), were scored on multiple representative sinus histology sections for each rabbit. The parameters were scored with zero, one, two or three to reflect whether changes between sides were absent, mild, moderate or severe.

### Bacterial Community Analysis

#### Sample Acquisition and DNA Extraction

Pairs of swabs were collected under endoscopic guidance from the middle meatus of each nostril at three time points (as indicated above). Swabs were placed in a nucleic acid preservative solution (RNAlater, Thermo Scientific, New Zealand) and put on ice immediately. In accordance with the manufacturer’s guidelines, samples were incubated with RNAlater for 24 h at 4°C before being stored at -20°C until further processing. DNA was extracted from the swabs using sterile Lysing Matrix E bead tubes (MP Biomedicals, Seven Hills, NSW, Australia) and the AllPrep DNA/RNA Mini Kit (Qiagen, Hilden, Germany), as described previously (Biswas et al., 2015). A negative DNA extraction control containing 200 μL sterile water was also carried out simultaneously.

#### Bacterial Community Sequencing

To evaluate bacterial communities the V3-V4 region of the bacterial 16S rRNA gene was amplified using 341F and 806R primers (Herlemann et al., 2011) together with Nextera DNA library prep kit adapters. PCR reactions and DNA purification were carried out as described elsewhere (Hoggard et al., 2017). In brief, approximately 100 ng of genomic template DNA was used in duplicate PCRs, each consisting of 35 cycles. Negative PCR controls were included in all PCR reactions as well as eluate from the negative extraction control, which yielded no detectable products. Amplicons from duplicate PCRs were pooled to a final volume of 50 uL and purified using Agencourt AMPure magnetic beads (Beckman Coulter Inc., USA). Purified PCR products were quantitatively assessed with Qubit dsDNA high-sensitivity kits (Life Technologies, New Zealand) and standardised to ~ five ng per sample. Purified products were submitted to Auckland Genomics Ltd for library preparation and sequencing using Illumina MiSeq (2 x 300 bp paired-end chemistry). Raw sequences are publicly available on the SRA-NCBI database (BioProject ID: PRJNA639396).

#### Bioinformatics

Raw sequences from 32 samples originating from six rabbits were merged and quality filtered using USEARCH (Edgar, 2010) version 11 with settings as previously described (Hoggard et al., 2017). For direct comparisons of bacterial community composition between a previous study and the current one, rabbit sinonasal swab samples from the previous study (Cho et al., 2018) were bioinformatically processed alongside the raw sequence reads obtained during our study. To account for differences in sequence length between the two studies, our merged sequences were trimmed to 256 bp, so as to overlap precisely the sequenced V4 16S rRNA gene region from the earlier study. Zero-radius operational taxonomic units (ZOTUs) representing 100% sequence similarity for each ZOTU were generated using the unoise3 algorithm within USEARCH (Edgar, 2016b). Each ZOTU was taxonomically assigned using the sintax classifier in USEARCH (Edgar, 2016a) with the RDP 16S rRNA gene database (version 16) (Cole et al., 2014). Sequences mapping to eukaryotic genomes were removed from subsequent analysis, and data were rarefied to an even sequencing depth of 2000 reads per sample. Analyses of bacterial community diversity as well as pairwise comparisons of single ZOTU abundances were conducted on samples of the left sinus only.

Alpha diversity (diversity within a sample) was calculated for Shannon and Simpson indices within the USEARCH pipeline. The Shannon and Simpson diversity metrics measure richness as well as evenness (relative abundance of ZOTUs and their distribution in a sample). Beta diversity (diversity between samples) was calculated as Bray-Curtis (BC) dissimilarity in R (version 3.6.0) using the vegdist command from the vegan package. The BC dissimilarity index quantifies the compositional similarity of the bacterial communities based on both presence/absence and the relative abundance of ZOTUs within the community.

### Statistical Analysis

Statistical analyses were carried out in R (version 3.6.0). Histological scores were evaluated using a paired t-test. Overall differences in alpha diversity were examined with an analysis of variance (ANOVA) followed by Tukey’s honest significant difference test. Permutational multivariate analyses of variance (PERMANOVA) based on BC distance matrices were conducted using the adonis command in the vegan package. A principal coordinate analysis (PCoA) was performed using the cmdscale command from the stats package for visualisation of the BC dissimilarity matrix. The resulting PCoA plot images the beta diversity data in two-dimensional space with samples that are more dissimilar being spaced further apart from each other. Pairwise comparisons of single ZOTU abundances were conducted using Kruskal-Wallis (KW) and *post-hoc* Dunn’s test with Benjamini-Hochberg adjustment for multiple comparisons.

## Results

### Establishing Sinonasal Inflammation

**Figure 2:**
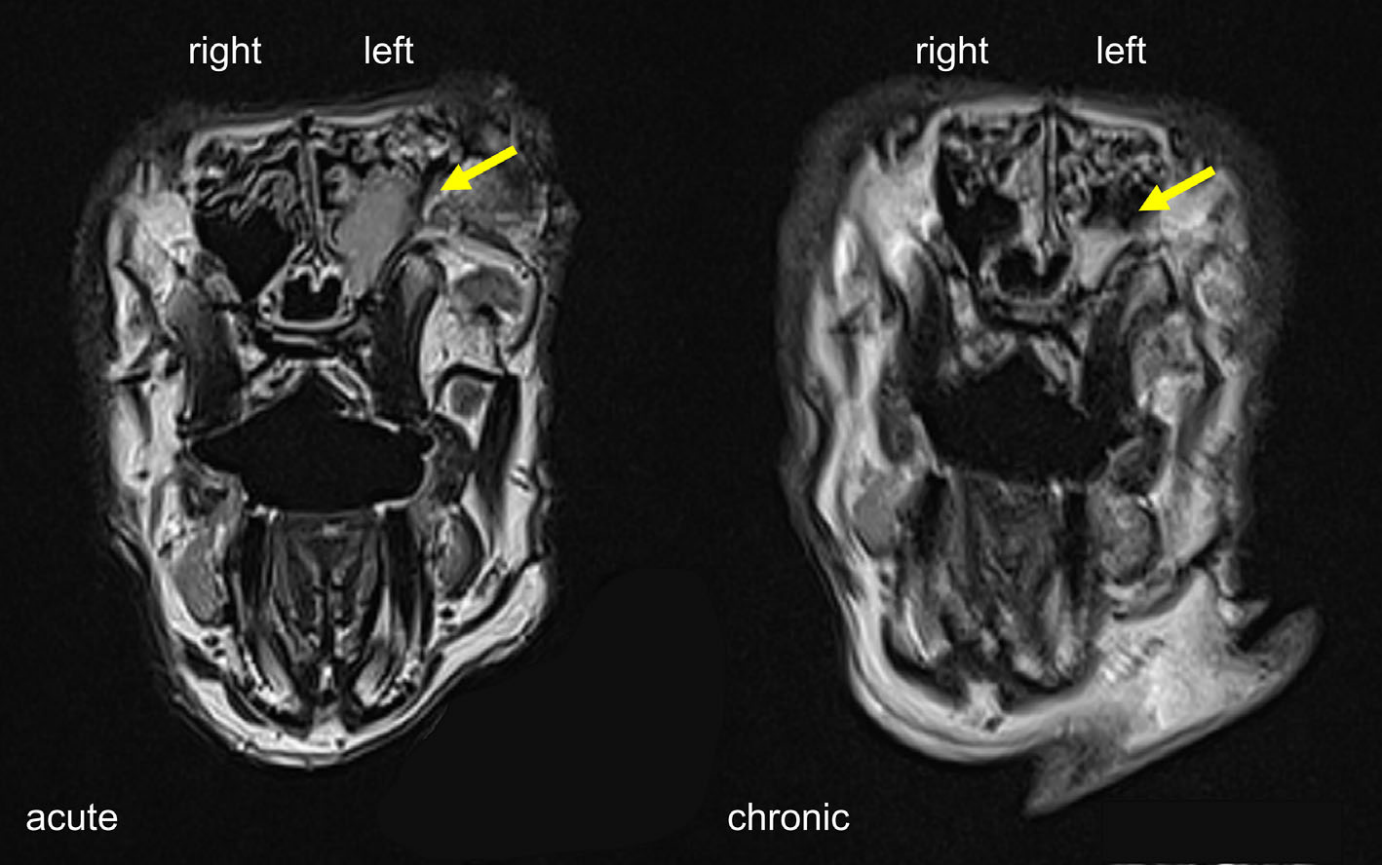
MRI scans of rabbit sinuses in coronal view. Severe and partial sinus opacification on the left side at the acute and chronic stage indicated by yellow arrows.

Two of the six rabbits (Rabbit #1 and #3) used in this study died from unknown cause during the post-sedation observation period after the surgical procedure. The first animal (#3) died eight days after Merocel^®^ placement and the second (#1) 24 h after Merocel^®^ removal. Sample collections were still performed from the first animal. Acute sinus inflammation was established in all six rabbits. Endoscopic examination showed moderate to severe sinus inflammation on the left (blocked) side of all rabbits at the acute stage (week four). Of note, endoscopy also revealed mild to moderate inflammation in the right (control) side at week four. At the chronic stage (week 14), obstructed sinuses remained mildly inflamed while all examined control sides presented normal (**Table 1**). MRI at week four showed unilaterally blocked sinuses in two rabbits, with one of them still showing partial opacification at week 14 (**Figure 2**). The overall success rate for establishing radiographic evidence of sinus opacification in the rabbits as determined by MRI at week 4 was 40% (2 out of 5 rabbits) and week 14, 25% (1 out of 4 animals). There was no evidence of opacification in the contralateral sinus at week 4 and week 14 in all animals.

**Table 1:** Endoscopic score grading for sinus inflammation.

rabbit #	Endoscopic scores					
	baseline		acute		chronic	
	left	right	left	right	left	right
1	0	0	3	2	NA	NA
2	0	0	2	1	1	0
3	0	0	NA	NA	NA	NA
4	0	0	2	1	NA	NA
5	0	0	3	1	1	0
6	0	0	2	1	NA	NA

0 = normal; 1 = mild inflammation; 2 = moderate inflammation; 3 = severe inflammation; 4 = severe inflammation with ulceration or polyps and luminal opacification.

Missing scores for animal #1 and #3 are due to premature death. Scores for animal #4 and #6 at the chronic stage were not recorded.

NA, not available.

### Histology

**Figure 3:**
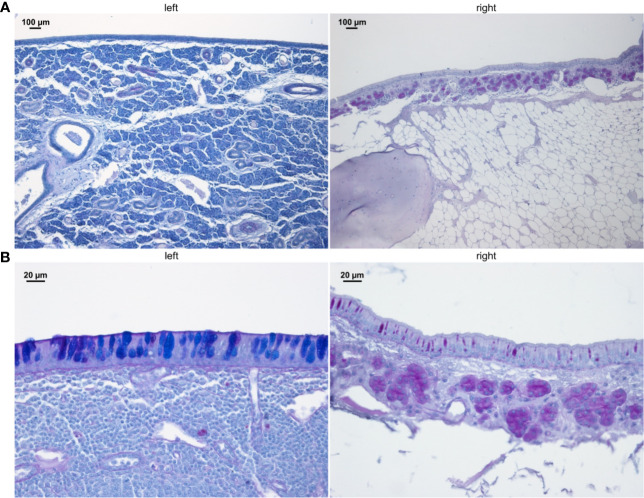
PAS staining of sinus mucosa showing signs of inflammation in the left (blocked) side and right (control) side at week 14. **(A)** Increased number of submucosal glands. **(B)** Goblet cell hyperplasia. Images are chosen exemplary and do not represent left and right sinus of one animal.

**Figure 4:**
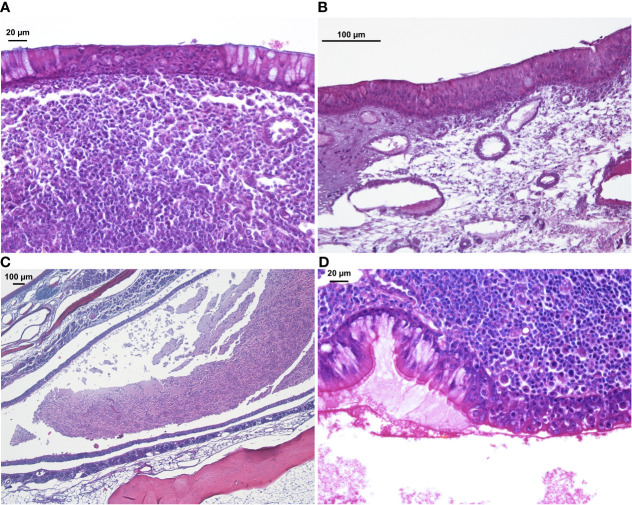
H&E staining of sinus mucosa showing signs of inflammation at week 14. **(A)** Epithelial infiltration of inflammatory cells. **(B)** Loose subepithelial matrix. **(C)** Luminal exudate. **(D)** Epithelial denudation.

**Figure 5:**
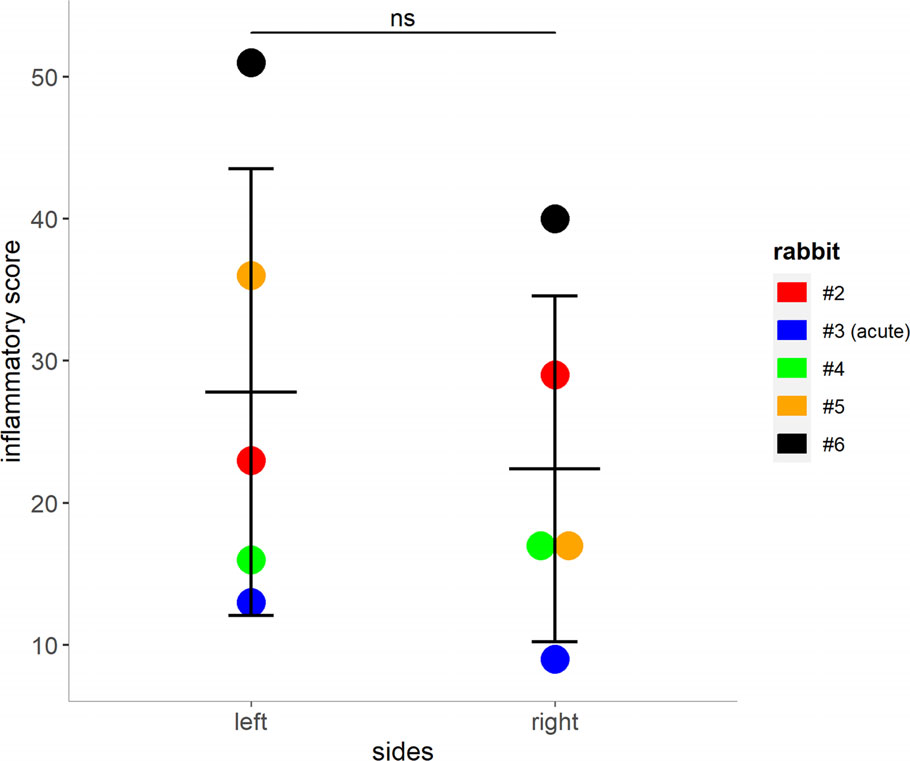
Mucosal score grading for rabbits 2 – 5. Higher scores indicate an increase of inflammatory markers within the left and right (control) sinuses of the rabbits (shown as mean + SD, paired t-test, p = 0.28). Tissue from rabbit #3 was harvested during the acute phase (day 8 post blockage) due to premature death. ns, not significant.

**Figure 6:**
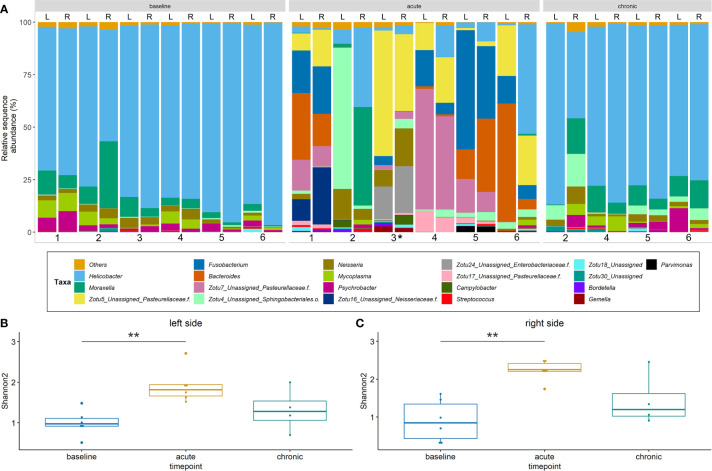
Bacterial community profiles and alpha diversity based on 16S rRNA gene sequence data. **(A)** Profiles are shown at genus level for the left (L) and right (R) side of rabbits’ sinuses. Samples missing at the chronic stage are due to the premature death of two animals. ZOTUs that could not be confidently assigned to genus level are presented as single ZOTUs with the next higher taxonomic classification. **(B) + (C)** Alpha diversity for the left and right sides. Shannon2 indices are shown for samples from different time points of the study. Significance levels: p<0.01 (**). *Samples from rabbit #3 were harvested on day 8 post blockage due to premature death.

The entire sinus mucosa was scanned on both sides for signs of inflammation. Some evidence of mucosal and submucosal inflammation was identified in all animals as shown in **Figures 3** and **4**: 1) hyperplasia of submucosal glands in the left sinuses (**Figure 3A**), 2) elevated number of goblet cells in the left sinuses (**Figure 3B**), 3) infiltration of inflammatory cells (**Figure 4A**), 4) loosening of the extracellular matrix or oedema (**Figure 4B**), 5) cellular exudate in the lumen (**Figure 4C**), and 6) epithelial denudation (**Figure 4D**). The histologic evidence of inflammatory processes was observed in the left (blocked) side as well as in the right (control) side of the same rabbit in all studied animals. Rabbits’ left sinuses had a higher score (mean +/- SD, 27.8 +/- 15.7) compared to the right side (22.4 +/- 12.2) even though the statistical significance was lacking (paired t-test, *p* = 0.28) (**Figure 5**). Rabbit #2, which had sinus opacification confirmed by MRI, has a higher score for the right (control) sinus. Rabbit #4 showed similar scores for each side, while rabbits #3, #5 and #6 had increased signs of inflammation in the left side, although MRI images showed clear sinuses. Even though there was only one sample (rabbit #3) from the acute stage, overall scores were higher at week 14 (chronic stage) than those at week 4 (acute).

#### Sinus Bacterial Community

Sequencing data from a total of 32 samples that were obtained from the left and right sinuses of 6 rabbits at baseline (n = 12), acute (n = 12) and chronic stage (n = 8) were evaluated. Data were stratified by animals for PERMANOVA which revealed that time point (i.e. baseline *vs.* acute *vs.* chronic) accounts for 48% of the observed variation (p < 0.001). Nostril side did not affect bacterial community structure significantly (*p* = 0.6). The baseline sinus microbiota was heavily dominated by the genus *Helicobacter* which, together with *Moraxella* and *Neisseria* (both at much lower relative abundances), accounted for 85 – 90% of the assigned sequences (**Figure 6A**). A significant compositional change was observed at the acute inflammatory stage compared to the baseline bacterial community. The abundance of ZOTUs assigned to several genera, including *Helicobacter*, *Moraxella* and *Neisseria* was significantly decreased while there was an apparent rise in the abundance of ZOTUs classified as *Bacteroides*, *Fusobacterium* and the family *Pasteurellaceae* (**Figure S1**). At the chronic stage, the sinus bacterial community returned to baseline composition within all sinuses. The overall number of taxa found in the left sinuses was significantly elevated at the acute inflammatory phase (**Figure 6B**). It is of note that alpha diversity patterns from the right (control) side (**Figure 6C**) closely mirrored those of the left side. Beta diversity analysis showed no differences between the bacterial communities in the left (blocked) and right (control) side at all time points (data not shown).

### Comparison With Bacterial Sequence Data From a Previously Published Study

16S rRNA gene amplicon data from 10 samples pertaining to the left sinuses of 10 rabbits (four from baseline, three from rabbits killed at week 4 and three from rabbits killed at week 14) from a previous study (Cho et al., 2018) were obtained to enable direct comparisons of bacterial community profiles between studies. Baseline bacterial communities in both studies were similar, with dominance by members of the genus *Helicobacter*. In the current study, *Moraxella, Mycoplasma* and *Neisseria* were the three most abundant genera after *Helicobacter*. While *Mycoplasma* was found to be the second most abundant genus, *Moraxella* and *Neisseria* were not amongst the 20 most abundant ZOTUS in the earlier study. At the acute stage, *Lactobacillus* and *Streptococcus* were the most abundant genera in the 2018 study, with a greater diversity of other, low-abundance taxa emerging in place of *Helicobacter*. In contrast to our findings, the previous study documented a persistently altered bacterial community at the chronic stage (**Figure S2**).

**Figure 7:**
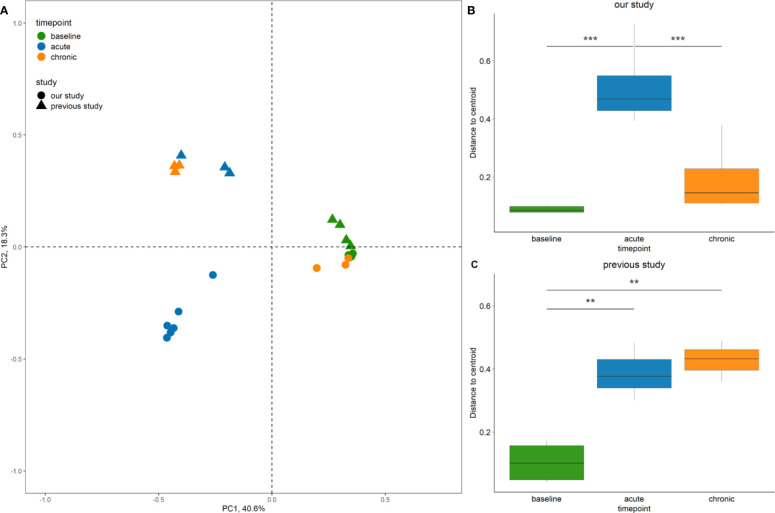
Comparison of bacterial community diversity between the study by Cho et al. (2018), and our study. **(A)** Principal Coordinate Analysis (PCoA) of sinus bacterial community structures at different time points. A two-dimensional PCoA plot was constructed based on Bray-Curtis dissimilarity. The time point of collection referring to baseline, acute and chronic inflammation is illustrated by colour, the origin of the sample from the 2018 or the current study is indicated by shape. Only rabbits left sides are shown. **(B, C)** Box plots indicating the distances of samples to the centroid. Asterisks indicate that the multivariate homogeneity of group dispersions with Tukey’s honest significant difference resulted in a significant difference. Significance levels: p < 0.01 (**), p < 0.001 (***).

Multidimensional scaling analysis reaffirmed our observation that the baseline composition of the bacterial communities in both studies were similar (i.e. close clustering of samples) (**Figure 7A**). By contrast, acute and chronic phase samples differed markedly between the two studies. Accordingly, persistent changes in beta diversity, as indicated by the distance of samples to the centroid, were observed for samples of the previous study (**Figure 7C**). In our study, group dispersion was significantly increased in samples from the acute phase only (**Figure 7B**).

## Discussion

In this study, we have replicated a recently established animal model that uses a period of middle meatal obstruction to cause unilateral sinusitis and assessed whether such obstruction could develop bilateral sinonasal inflammation in an animal model. To our limited knowledge, this is the first study to demonstrate that unilateral sinus blockage can develop inflammation in the contralateral side in an animal model. Obstruction resulted in acute sinusitis and three out of four rabbits did have increased signs of bilateral inflammation histologically at week 14. However, persistent radiologic inflammatory signs were only seen in one out of four rabbits that reached the endpoint of the study (eight weeks after removal of obstruction).

### Acute *Versus* Chronic Sinusitis

We have found that the model is excellent for the study of acute inflammation but challenging for the study of long-term inflammatory changes that would be required to model CRS. Endoscopic but not radiologic findings consistent with acute sinusitis were observed in all study animals after four weeks of blockage in the treated middle meatus. However, success in establishing long term mucosal changes in these animals, all of which were New Zealand white rabbits, differed between the two studies. We hypothesise that this discrepancy may be attributed to any or some of the differences between the two studies: anaesthetic procedure (injectable only in the previous study *vs.* injectable and gaseous in the current study), source of animals (lab bred (*Pasteurellaceae* free) *vs.* farm raised), specimen collecting sites/process, induction of acute sinusitis with full opacification and weight (2-4 kg *vs.* 4-6 kg) of the rabbits. Hunter and colleagues investigated the role of airway mucus as a nutrient source (e.g., short chain fatty acids) in stimulating the growth of putative pathogens (e.g., *Pseudomonas* spp.) in the cystic fibrosis airway (Flynn et al., 2016). This study demonstrated that potential pathogens (e.g., *Staphylococcus*, *Pseudomonas*), that cannot utilize the mucin, do not establish an airway infection until anaerobic, mucin-fermenting bacteria have colonized. Therefore, it seems crucial to generate an anaerobic sinus cavity filled with pathogenic mucus during the acute sinusitis period to induce chronic inflammatory features later in this animal model. Furthermore, subtle differences could have arisen due to the study being performed by another research team and at a different location, resulting in an altered environment for the rabbits.

It is also of note that radiologic imaging in this study was done by MRI while the previous study used micro-computed tomography (µCT). The latter is the preferred method to measure sinusitis in rabbit models and could be more sensitive in detecting sinus opacification (Kerschner et al., 2000). In addition, Cho et al. (2018) reported sinus opacifications in all rabbits during the acute phase when assessed using high resolution µCT. There was a discrepancy between radiology (MRI) and histology findings in our study and MRI results did not reflect the subtle histologic changes in the sinus mucosa. Similarly, when Ozan *et al.* inoculated *Staphylococcus aureus* in a rabbit model, the histopathological and CT findings in experimental rabbits were not correlated (Ozcan et al., 2011).

Histological findings as previously described (Cho et al., 2018) could be reproduced in our model, however results were rather inconsistent and were not always concordant with macroscopic observations. It is of note that highly variable changes in the mucosa could be found within the same side in a single histological section. This highlights the difficulty with histopathological examination of the sinonasal mucosa for signs of chronic inflammation, and the requirement to examine multiple sections of several different sites within a sinus (Jacob et al., 2001). Local variation in inflammatory changes throughout the sinus cavities has been observed in CRS patients, in whom certain areas of the sinus mucosa can be highly inflamed while others show little signs of inflammation (Suh and Kennedy, 2011).

The microbiome results are in accordance with the results of the previously published report of this model, with an increase in bacterial diversity at the acute sinusitis phase (Cho et al., 2018). However, results in the two studies differed at the chronic stage. We suspect that the distinct bacterial community profiles reflect the contrasting inflammatory states in the two studies in association with the different immunologic patterns of farm-raised versus lab-bred animals. In this rhinogenic sinusitis model, inflammation is initiated from the impairment of the natural sinus function. Thus, the observed microbiological changes more accurately represent a physiologic response of the sinus microbiome when compared to bacterial inoculation models.

### Bilateral Sinus Inflammation From Unilateral Sinus Outflow Obstruction

We found in our pilot study that extending the period of sinus outflow obstruction from two to four weeks resulted in increased inflammation. The more severe sinusitis on the obstructed side appears to lead to inflammation on the contralateral (control) side which was associated with significant changes to the sinus microbiota at week 4 (acute stage). In comparison, the previous description of this model reported consistent inflammation and opacification of all obstructed sinuses at two weeks which persisted for another 12 weeks while contralateral sinuses remained clear (Cho et al., 2018). However, the bacterial community profiles of the contralateral (control) side were not investigated in that study.

The study by Perloff et al. clearly demonstrated an extensive inflammatory involvement extending from the infected sinus to the bone in rabbits infected with *P. aeruginosa* (Perloff et al., 2000). In this study, the changes in the microbiota during the acute phase (an increase in anaerobic bacteria) could initiate the inflammatory process noted in the chronic phase, even though the bacterial community composition had returned to baseline by that time (week 14). CRS generally presents bilaterally and if the potential for inflammation to spread locally is also present in human patients, it could explain some of the clinical observations. One of the major advantages of creating a unilateral model is that the contralateral side can be used as a control (Lux et al., 2019). Considering these findings, however, we advise careful monitoring of the mucosa and microbiota of the contralateral sinus and recommend that the contralateral sinus may not be used as a true negative control.

The interaction between both sinuses observed in this study may be triggered by neural and vascular reflexes and warrants further investigation. The described model provides the basis to study these inflammatory pathways on a molecular level. In addition, the release of systemic inflammatory mediators as result of the unilateral intervention may promote contralateral sinus inflammation. Specifically, the examination of cytokine patterns of the tissue inflammation in the obstructed and unobstructed side in future studies may help reveal potential implications for CRS.

### Host Response in Rabbits

Farm-raised or wild rodents have immune responses that are different to those of laboratory-bred animals (Abolins et al., 2017). While antibody responses are elevated in wild animals due to more extensive antigenic challenges, other immunologic processes such as proliferation and cytokine release are depressed (Abolins et al., 2017; Viney and Riley, 2017). The free-living rabbits used in our study appear to have a more competent immune system that can maintain immune homeostasis. Therefore, immunopathology is minimised, and acute inflammation is resolved rather than progressing to a chronic inflammatory state. Reduced immune cell proliferation and cytokine response may contribute to the variable histological findings in this study.

There are other limitations when drawing conclusions about human pathophysiology from animal findings. The previous study found that the dominant bacterial phyla in the rabbit model were the same as those in human sinuses. In the current study, a genus-level taxonomic classification was applied which revealed that the most abundant bacterial genus (*Helicobacter*) is not commonly found in human sinuses. However, putative disease-associated genera (e.g. *Streptococcus*) remain the same between hosts. Despite different sinonasal bacterial community profiles in rabbits and humans, observing shifts in the microbial community throughout the inflammatory process can provide helpful insights into the pathogenesis of CRS in human patients.

## Conclusion

This study reaffirmed the ability to develop sinusitis in a rabbit model without inoculation of any pathogens as previously described (Cho et al., 2018). Even though it is technically challenging to generate a persistent sinus mucosal inflammation, this rabbit model seems to be reproducible and provides a potential for studying host-microbe interactions during sinonasal inflammation under a high level of experimental control. We further demonstrated that bilateral sinonasal inflammation can be caused by inducing unilateral sinus blockage. Further research is necessary to investigate this novel observation and its potential implication for the clinical observations seen in CRS.

## Data Availability Statement

The data presented in the study are deposited in the SRA NCBI repository, accession numbers SRR12012423 - SRR12012402.

## Ethics Statement

The animal study was reviewed and approved by University of Auckland Animal Ethics Committee (AEC# 001910).

## Author Contributions

CL, KB, RD and MT planned and conceived the study. CL carried out the experiments, data analysis and took the lead in writing the manuscript with support from KB, RD, MT and D-YC. JJ and CD helped to perform the surgical procedures. SW-T helped to carry out the histological experiments and analysis. All authors contributed to the article and approved the submitted version.

## Funding

CL, RD and MT were supported by funding from the Garnett Passe and Rodney Williams Memorial Foundation (91073712790). D-YC received funding from the NIH/National Institutes of Allergy and Infectious disease (K08AI146220), the Triological Society Career Development Award 2019 and the Cystic Fibrosis Foundation K08 Boost Award (CHO20A0-KB).

## Conflict of Interest

The authors declare that the research was conducted in the absence of any commercial or financial relationships that could be construed as a potential conflict of interest.

## Publisher’s Note

All claims expressed in this article are solely those of the authors and do not necessarily represent those of their affiliated organizations, or those of the publisher, the editors and the reviewers. Any product that may be evaluated in this article, or claim that may be made by its manufacturer, is not guaranteed or endorsed by the publisher.

## Supplementary Material

Supplementary Figure 1 Pairwise comparisons of relative sequence abundances between timepoints. Selected single ZOTUs are shown. Significance levels: p<0.05 (*), p<0.01 (**).

Supplementary Figure 2 Bacterial community profiles of rabbits from the 2018 study. Profiles are shown at genus level . Only the left side was sampled from different individuals at each time point. In contrast to our results, the decreased abundance of Helicobacter is compensated by a large variety of “other” genera (orange portion of stacked bars).
