# Supplementary figures and images for: Unilateral Intervention in the Sinuses of Rabbits Induces Bilateral Inflammatory and Microbial Changes
Source: Front Cell Infect Microbiol. 2021 Sep 14;11:585625. doi: 10.3389/fcimb.2021.585625 (PMC8477012; doi:10.3389/fcimb.2021.585625)

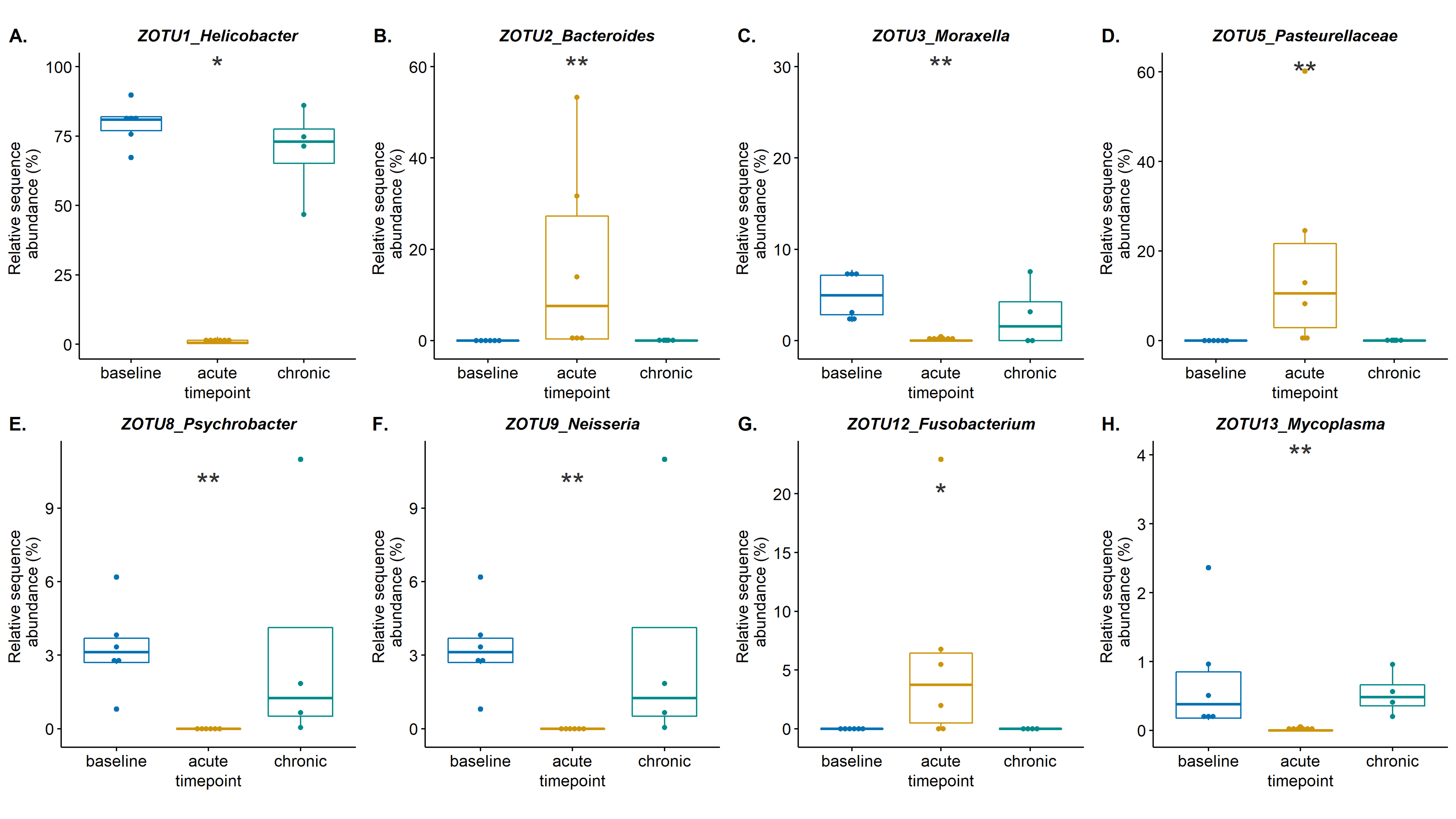

Supplement: Supplementary Figure 1 — Pairwise comparisons of relative sequence abundances between timepoints. Selected single ZOTUs are shown. Significance levels: p<0.05 (*), p<0.01 (**). [file Image_1.tif]

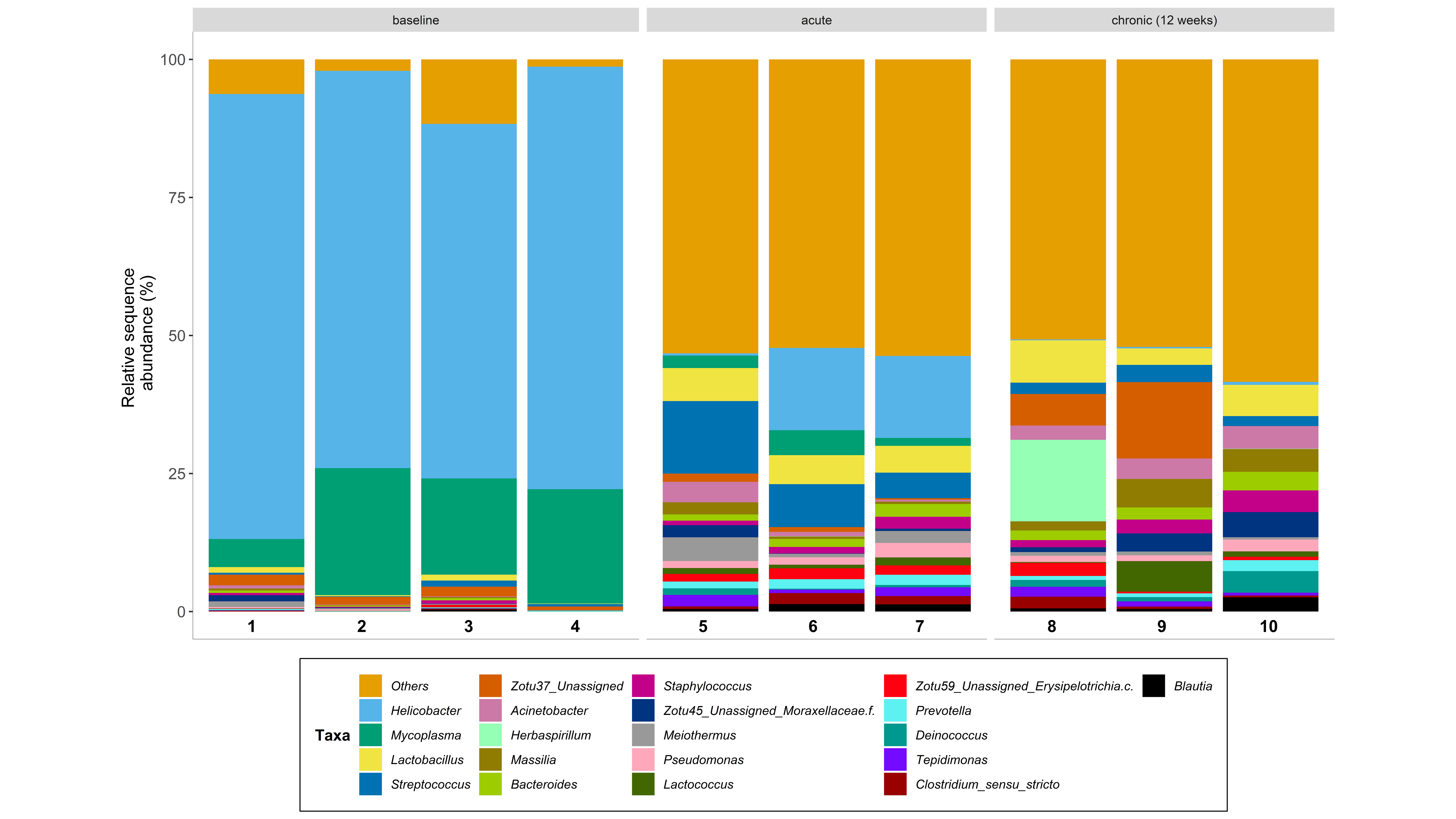

Supplement: Supplementary Figure 2 — Bacterial community profiles of rabbits from the 2018 study. Profiles are shown at genus level . Only the left side was sampled from different individuals at each time point. In contrast to our results, the decreased abundance of Helicobacter is compensated by a large variety of “other” genera (orange portion of stacked bars). [file Image_2.tif]
