# Supplementary material for: Unilateral Intervention in the Sinuses of Rabbits Induces Bilateral Inflammatory and Microbial Changes
Source: Front Cell Infect Microbiol. 2021 Sep 14;11:585625. doi: 10.3389/fcimb.2021.585625 (PMC8477012; doi:10.3389/fcimb.2021.585625)
Supplement: Supplementary file 3 [file Table_1.docx]

**Table S1. Parameters for the description of mucosal inflammation and integrity**

| epithelium | base membrane | submucosa |
| --- | --- | --- |
| denudation | thickness | thickness |
| hypertrophy | inflammatory cells | extracellular matrix |
| goblet cell hyperplasia |  | inflammatory cells |
| inflammatory cells |  | mucus glands |
| mucus production |  |  |
| shedding |  |  |
